# Flowering and Flower Maturation in Valley Oak *Quercus lobata*

**DOI:** 10.1002/ece3.73720

**Published:** 2026-05-30

**Authors:** Walter D. Koenig, Ian S. Pearse, Mario B. Pesendorfer, William J. Carmen, Johannes M. H. Knops

**Affiliations:** ^1^ Hastings Reservation University of California Berkeley Carmel Valley California USA; ^2^ U.S. Geological Survey Fort Collins Science Center Fort Collins Colorado USA; ^3^ Department of Ecosystem Management, Climate, and Biodiversity, Institute of Forest Ecology BOKU University Vienna Austria; ^4^ Carmen Ecological Consulting Mill Valley California USA; ^5^ School of Biological Sciences University of Nebraska Lincoln Nebraska USA

**Keywords:** acorn production, catkins, life‐history tradeoffs, masting behavior, pollen coupling, pollen limitation, *Quercus lobata*

## Abstract

We investigated factors driving flowering and flower maturation and their effects on acorn production at both the annual and individual trees levels in a population of valley oak ( *Quercus lobata* ) in central coastal California, USA. Among years, female flower production was negatively correlated with rainfall, while warm spring temperatures and a large acorn crop the previous year decreased the proportion of flowers maturing into acorns. Among trees, individuals with greater access to ground water growing in warmer microclimates produced more catkins and more female flowers. In addition, male and female flower abundance were positively correlated even after controlling for tree size and resources, countering the hypothesis of a life‐history tradeoff between investment in male and female flowering. Factors limiting pollen availability include weather‐driven effects (both the pollination Moran effect and environmental vetos), phenological synchrony, and pollen coupling, factors that were difficult to distinguish. All three flower indices explained a significant proportion of variance in acorn production at both the annual and individual tree levels. Most important, however, was variation in female flower maturation, while both female and male flower abundance explained between one‐quarter and one‐third of variance in acorn production. *Quercus lobata* is not only a flower maturation masting species, but also a female flower and male flower masting species. The underlying drivers of flower initiation, fertilization, and flower maturation yield important insights into the factors influencing masting behavior in perennial plants and represent an area that can benefit from more empirical study.

## Graphical abstract

We investigated the factors influencing flower production both among years and among individual trees of the valley oak *Quercus lobata* . Among years, weather conditions, particularly winter rainfall, drive female flower production, while among trees, male and female flowers were positively correlated, countering the hypothesis of a life‐history trade‐off between them. Variation in acorn production among individuals is primarily explained by female flower production, while half the variation among years is explained by differences in flower maturation and the rest by differences in female and male flower production.

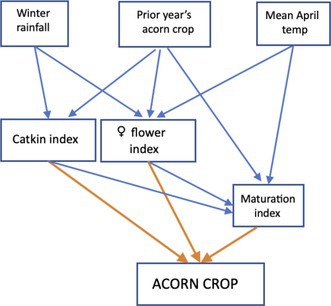

## 1 Introduction

Masting—variable and synchronized reproduction by a population of plants—is a phenomenon widespread among temperate and boreal trees that has considerable importance to forest ecosystems (Ostfeld and Keesing 2000; Kelly and Sork 2002; Pearse et al. 2016; Bogdziewicz, Ascoli, et al. 2020). The evolutionary drivers of masting are believed to be an economy of scale by which reproduction is more efficient when consolidated into larger episodes. The two most general economies of scale driving masting behavior are predator satiation and pollination efficiency (Norton and Kelly 1988; Kelly 1994). The latter—increased pollination efficiency—is thought to be particularly important in wind‐pollinated, self‐incompatible species such as oaks (*Quercus* spp.) in which both male and female flower investment is required at the population level, at least in mast years (Satake and Iwasa 2000; Crone and Rapp 2014; Pearse et al. 2015). Pollination efficiency is also important to masting at the proximate, mechanistic level, both as a driver of synchrony and annual seed‐crop variability due to pollination failure in some years (Satake and Iwasa 2002a, 2002b; Pearse et al. 2016).

Given the multiple roles that pollination efficiency potentially plays, interest in the flowering behavior of masting trees has grown in the last two decades from being controversial as to whether pollen limitation occurs in wind‐pollinated species (Koenig and Ashley 2003) to studies investigating the mechanisms by which pollen limitation acts (Fleurot et al. 2024; Crone and Rapp 2025).

Beause of the need for cross‐pollination, isolated trees of self‐incompatible species may suffer pollen limitation in fragmented landscapes independent of environmental factors (Knapp et al. 2001; Pearse et al. 2026). In general, however, weather is key to most hypotheses of pollination efficiency. Weather may act directly as an “environmental veto” by freezing female flowers (Pearse et al. 2016; Bogdziewicz et al. 2018; Bogdziewicz, Żywiec, et al. 2019; Baumgarten et al. 2023) or via a “pollination Moran effect” by affecting pollen flow. Examples of the latter include rain, high humidity, or bad timing, any of which can impede pollen flow (Cecich and Sullivan 1999; García‐Mozo et al. 2007; Schermer et al. 2020).

Weather also affects pollen availability indirectly. The “phenological synchrony” hypothesis, for example, proposes that the synchrony of flowering among trees within a population, driven by environmental conditions, affects pollen availability and thus masting behavior (Koenig et al. 2015; Bogdziewicz, Pesendorfer, et al. 2020). The timing of flowering by individual trees relative to the local population also affects pollen availability and seed production (Koenig et al. 2012), as can the coordination of male and female flower production among trees in the population via the phenomenon of “pollen coupling” (Isagi et al. 1997; Kelly et al. 2001; Crone and Rapp 2014).

Weather conditions have additional effects through their influence on the internal resource dynamics of trees that are the basis of “resource budget” models of masting (Isagi et al. 1997; Rees et al. 2002; Crone et al. 2009; Crone and Rapp 2014). Although such models remain largely theoretical, ongoing work investigating the genetics of masting promises to provide links between resources and the physiological basis of masting, including how weather factors influence floral development and maturation (Satake and Kelly 2021).

Prior work on the endemic California valley oak ( *Quercus lobata* ) has found both empirical and experimental support for the importance of pollen limitation as a driver of variable and synchronous acorn production (Koenig et al. 2012, 2015; Pearse et al. 2015, 2026; Pesendorfer et al. 2016). Other studies based on litter traps placed under individual trees of three oak species (including *Q. lobata* ) over a period of 5 years concluded that an inverse relationship between male and female investment within trees was a byproduct of relatively constant investment in male reproduction combined with a positive correlation with the far greater investment in female function—mostly acorns—rather than a tradeoff between the two (Knops and Koenig 2012). Here we expand on these prior studies with an analysis of male and female flower dynamics and their effects on both year‐to‐year acorn fluctuations and variation in acorn production among trees within a single population. Our goals are to better understand the factors affecting flowering and pollen production, consider mechanisms by which pollination efficiency is important, and determine the relative importance of different aspects of flowering dynamics to subsequent acorn production.

## 2 Methods

### 2.1 Study Area and Species

This work is part of a long‐term study of acorn production at Hastings Natural History Reservation (henceforth “Hastings”) in central coastal California (36.38^o^N, 121.56^o^W) initiated in 1980 (Koenig, Mumme, et al. 1994; Koenig et al. 2015, 2020). The study involves 88 *Q. lobata* , of which 81 were still alive as of 2024. Like all true oaks, *Q. lobata* is wind pollinated, and, as a member of the *Quercus* section of the genus *Quercus* (Denk et al. 2017), matures acorns in a single year. It is self‐incompatible (Sork et al. 2002; Pearse et al. 2015), and a large proportion of ovules are pollinated by pollen from trees > 200 m away (Abraham et al. 2011).

### 2.2 Flower and Acorn Production

We quantified catkins, female flowers, and acorns on individual trees. Catkin abundance was estimated each year from 2003 to 2025 (*N* = 22 years; 2023 was missed) by visiting each tree weekly for budburst starting between late January and 1 March, depending on the year. Once budburst occurred, we categorized the abundance of catkins at each weekly visit on a scale of 0–3, where 0 = no catkins; 1 = a few catkins; 2 = an intermediate number of catkins; and 3 = abundant catkins. We then determined the maximum estimated abundance of catkins recorded for each tree each year, referred to as the “catkin index.”

Female flowers and their maturation into acorns were quantified using marked branches 2009–2018 (*N* = 10 years). For each tree, two branches were arbitrarily chosen and tagged. In late spring (May–June) we counted the number of female flowers per branch, and in September we counted the number of acorns on the same branches. Female flower abundance per 100 leaves (the “female flower index”) was our standardized measure of female flower abundance. Our measure of how efficiently female flowers matured into acorns was the number of acorns per female flower on the branches (the “flower maturation index”) (Pearse et al. 2015). The catkin, female flower, and flower maturation indices are referred to together as the “flower indices.”

The overall acorn crop of each tree was quantified in September by means of visual surveys in which two observers scanned different parts of the canopy and counted as many acorns as they could in 15 s (Koenig, Knops, et al. 1994; Koenig, Mumme, et al. 1994). Counts were added and *ln*‐transformed to reduce non‐normality. Tree size was determined by diameter at breast height (DBH) measured in September 2008.

### 2.3 Environmental Conditions

Temperature and rainfall were measured at the Hastings weather station located within 3.5 km of all trees. The microclimate of individual trees was measured using small, automated temperature recorders (iButtons; Maxim Integrated Products, Sunnyvale, CA) located ~1.5 m above ground on the north side of each tree (Koenig et al. 2015). iButtons were programmed to record temperatures at 4‐h intervals starting at 04:00 each day; the 08:00 temperature reading was excluded because not all iButtons were shaded at that time of day.

### 2.4 Water Availability and Nutrients

Key factors potentially affecting flowering and reproduction include tree size (Zhang and Jiang 2002; Masaka and Takada 2006), access to ground water (Hanes 1965; Knops et al. 2007; Barringer et al. 2013), foliar nutrients (Güsewell 2004; Sardans et al. 2012; Han et al. 2014; Fernández‐Martinez et al. 2019), and prior reproductive effort (Barringer et al. 2013). Size of the trees was estimated by diameter at breast height (DBH) measured in 2008. Access to ground water was measured by means of xylem water potential (XWP) using the pressure chamber technique (Waring and Cleary 1967) in September 1991 (Knops and Koenig 1994). XWP values vary from year to year depending on rainfall but are concordant among trees (Knops and Koenig 2000). Thus, although XWP of trees was only measured once, values provide an index of relative water availability across trees.

Foliar total nitrogen (N) and foliar total phosphorus (P) were measured from leaves collected 7–8 July 1992 and again 10–15 June 2024 on the outer canopy of each tree. Leaves collected in 1992 were analyzed for total nitrogen and total phosphorus on a continuous flow autoanalyzer using standard procedures (Knops 1994). Leaves collected in 2024 were analyzed for total nitrogen by means of flash combustion (AOAC Official Method 972.43 2006) and for total phosphorus by nitric acid/hydrogen peroxide microwave digestion (Meyer and Keliher 1992; Sah and Miller 1992) at the University of California, Davis, Analytical Laboratory. Pearson correlations between both foliar N and foliar P sampled in 1992 and 2024 were both highly significant (foliar N: *r* = 0.42, *df =* 78, *p* = 0.0001; foliar P: *r* = 0.57, *df =* 78, *p* < 0.0001). There were also no significant differences in their means (Wilcoxon two‐sample tests, foliar N: W = 9, *p* = 0.89; foliar P: W = 6.5, *p* = 0.77). Consequently, we used the mean of the two values in our models.

### 2.5 Analyses

#### 2.5.1 Statistical Analyses

Statistics were performed using R 4.4.2 (R Core Team 2024). *p* < 0.05 was the cutoff for determining statistical significance. We used linear regressions to test for a tradeoff between investment in catkins and female flowers controlling for tree size and three indices of resource availability (XWP, foliar N, and foliar P), and to estimate the proportion of total variance in acorn production explained by the flower indices.

Analyses investigating factors affecting flower abundance and how flowering translated into the acorn crop were conducted by means of path analysis with the *lavaan* package (Rosseel 2012). Analyses were done both at the annual level (mean flowering and acorn production over all trees for the year) and at the individual tree level (mean flowering and acorn production overall years for each tree). Estimated effect sizes are illustrated using standardized values to allow comparison among explanatory variables; unstandardized values and their standard errors are listed in Table S1. Only the 10 years (2009–2018) for which we quantified both catkins and female flowers were used in the path analyses.

#### 2.5.2 Factors Affecting Flowering

The general factors driving flowering dynamics are resources and weather. They are not distinct, as weather influences many of the resources potentially important to plants. We consider them separately, although we recognize that it is not currently possible to disentangle their effects in most cases.

For both the annual and individual tree levels, path analyses were arranged at three (partially overlapping) levels (Figures 2a and 3a). At the base was the mean size of the overall acorn crop for the year (at the annual level) and the mean productivity of the tree (at the individual level). Because our main interest was the effects of flowering and flower maturation on the acorn crop, paths leading to the acorn crop included only the three flower indices (Pesendorfer et al. 2016; Bogdziewicz, Szymkowiak, et al. 2019). Catkin abundance, a clear factor potentially affecting the acorn crop at the annual level, was also included in the individual tree analysis despite self‐incompatibility because catkins require resources that could otherwise go elsewhere. At the top level were the various factors, both environmental and resource‐related, potentially driving the flower indices.

At the annual level (Figure 2a), winter rainfall (1 October of the prior year through 31 March) was considered a potential driver of the catkin and female flower indices and mean April temperature of the female flower and maturation indices. Both were measured at Hastings Reservation headquarters. The prior year's acorn crop, an index of prior resource depletion, was considered a potential driver of all three flower indices. The justification for this is the negative lag‐1 correlation in acorn production by *Q. lobata* (Koenig, Mumme, et al. 1994; Barringer et al. 2013) and in seed production by many other masting species (Koenig and Knops 2000). Over the 46‐year period (1980–2025) that we have surveyed *Q. lobata* at Hastings, the lag‐1 autocorrelation of the mean annual (*ln*‐transformed) acorn crop was −0.512 (*p* < 0.001).

In addition, the male and female flower indices were considered potential factors affecting flower maturation. Timing of the two weather variables was determined by prior work (Koenig et al. 2020) and the observation that flowering usually takes place between late Feburary to early May. We used rainfall, but not temperature, because of the importance of water availability to trees and because it is inversely correlated with temperatures during the winter.

At the individual tree level, mean winter (as defined above) and mean April temperatures, measured by the iButtons at each tree, were considered potential drivers of the catkin index (winter temperature), the maturation index (April temperature), and the female flower index (both mean winter and mean April temperatures). Values were averaged over the 10 years for which we had data on both catkins and female flowering. Resources measured at individual trees included leaf nutrients (foliar N and foliar P), access to ground water (XWP), and radial size (DBH). Although interactions between some of the resource variables are possible, the relatively small sample sizes precluded including them in the path analyses.

## 3 Results

### 3.1 Annual Variation

**FIGURE 1:**
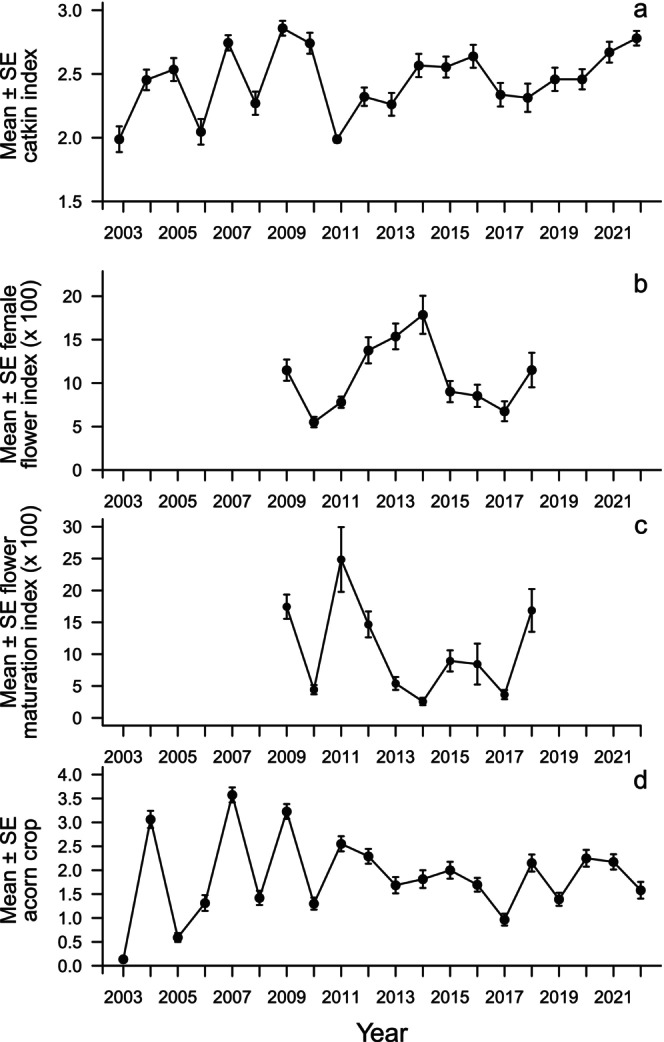
Annual mean (± standard error [SE]) across all *Quercus lobata* of their (a) catkin index (2003–2022), (b) female flower index (×100) (2009–2018), (c) female flower maturation index (×100) (2003–2022), and (d) *ln*‐transformed overall acorn crop from the visual surveys (2003–2022).

Annual variability in mean flowering and the mean (*ln*‐transformed) acorn crop is illustrated in Figure 1. *Quercus lobata* is a moderate masting species (Koenig et al. 1996). Across all years for which we have data (1980–2025; *N* = 46 years) the coefficient of variation (CV = standard deviation/mean × 100) of annual acorn production was 49.8 using the *ln*‐transformed mean values and 85.8 using the untransformed survey counts.

### 3.2 Flower Dynamics

**FIGURE 2:**
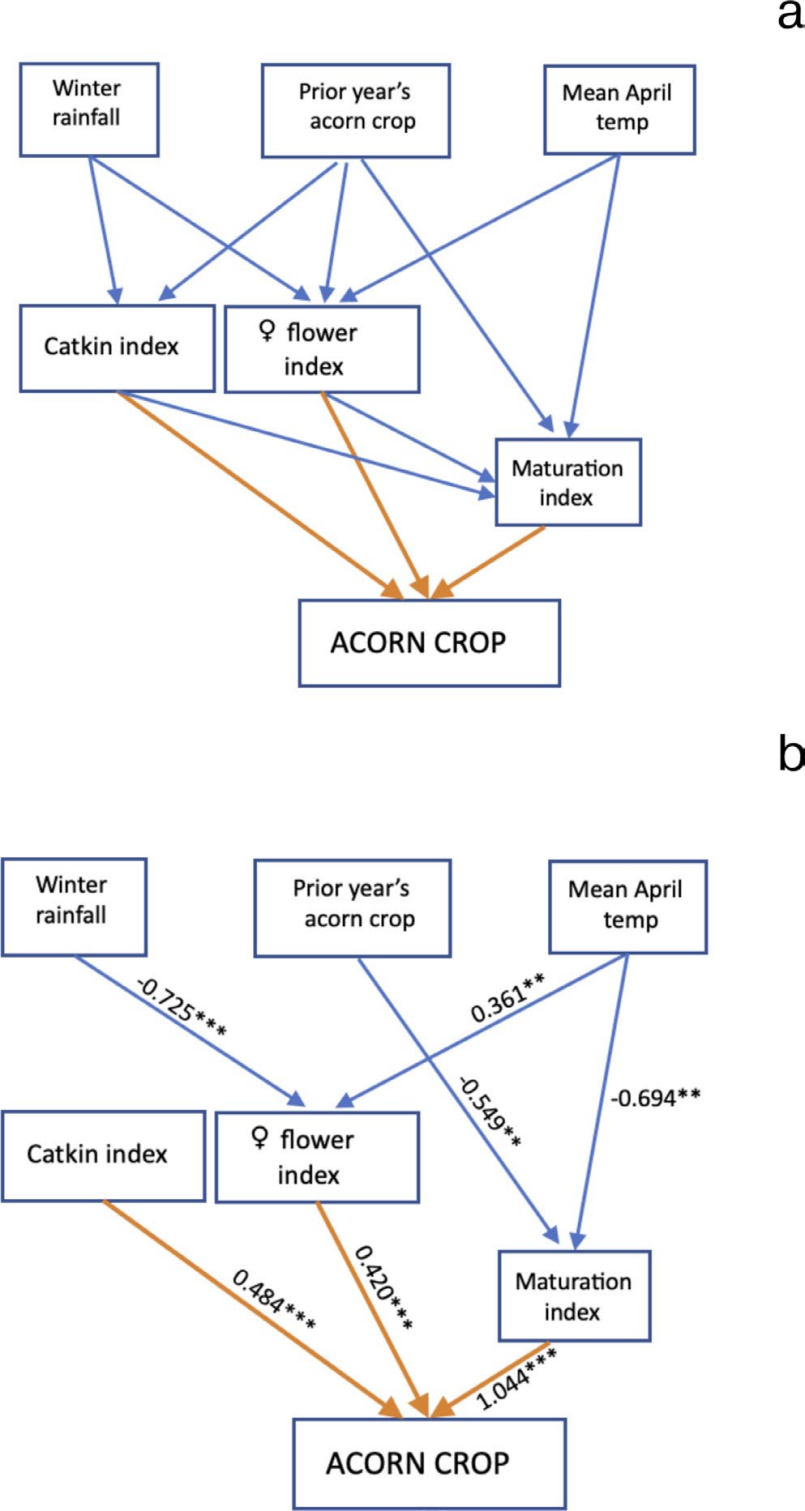
(a) The a priori causal relationships tested by means of path analysis for mean annual differences in *Quercus lobata* acorn production. (b) The path diagram with nonsignificant effects removed. Standardized effect sizes are listed. ***p < 0.001; **p < 0.01; other *p* > 0.05. *N* = 10 years. For complete details, see Table S1.

The a priori causal links in the path analyses are illustrated in Figure 2a (annual level) and 3a (individual tree level). However, preliminary analyses indicated that neither of the foliar nutrients nor tree size (DBH) significantly affected any of the three flower indices, and they were thus, although illustrated, not included in the final model of individual tree flowering.

**FIGURE 3:**
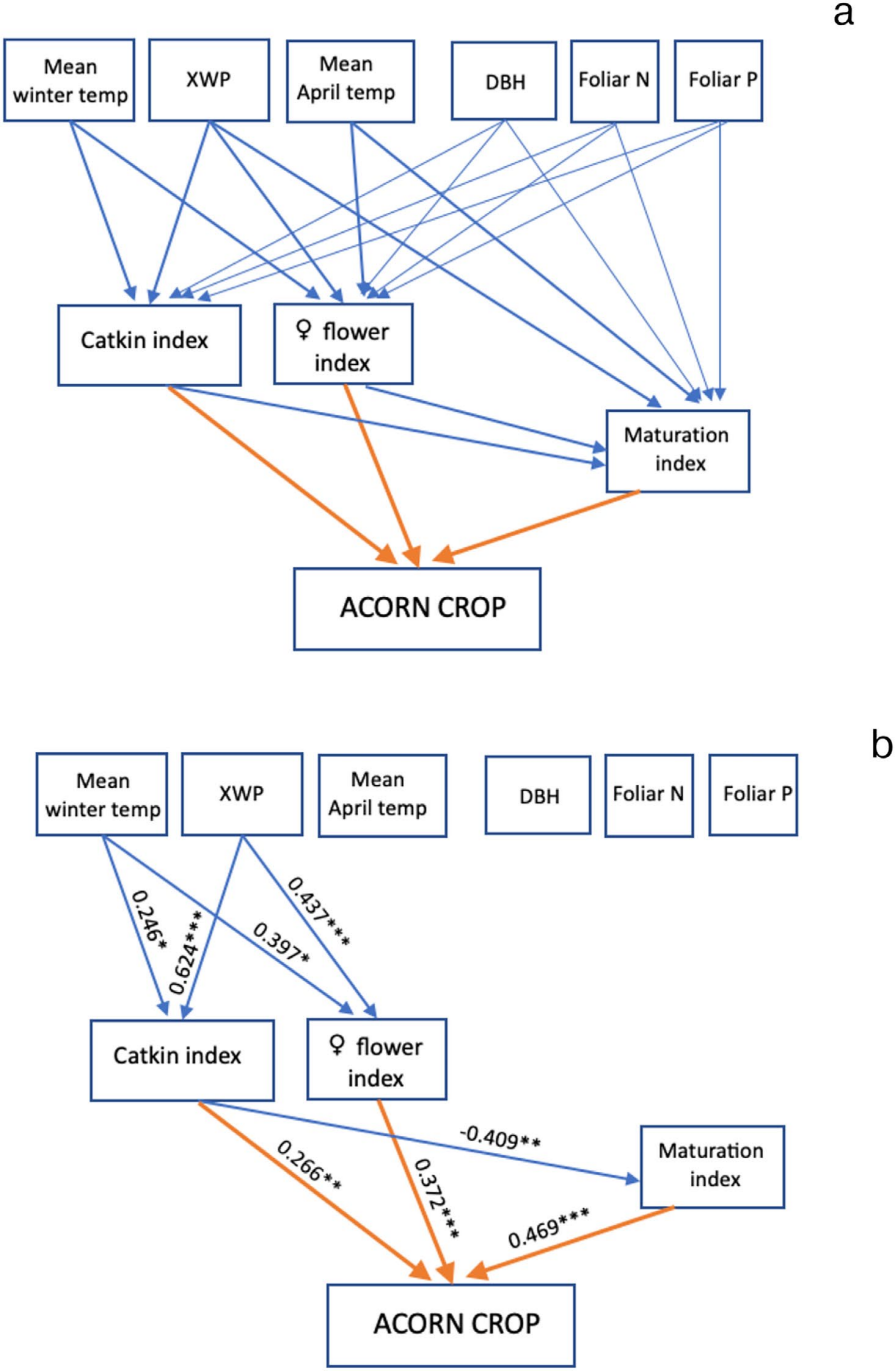
(a) The a priori causal relationships tested by means of path analysis for mean differences in acorn production among individual *Quercus lobata* . (b) The path diagram with nonsignificant effects removed. (The two shaded variables were not included in the final model.) Standardized effect sizes are listed; ***p < 0.001; **p < 0.01; *p < 0.05; other *p* > 0.05. *N* = 69 trees. For complete details, see Table S2.

Figures 2b and 3b illustrate the models with (only) significant interactions denoted by their standardized estimated effect sizes and levels of significance; full results are listed in Tables S1 and S2. At the annual level (Figure 2b), neither winter rainfall nor the prior year's acorn crop affected catkin production. Female flower production, however, was inhibited by winter rainfall and positively related to mean April temperature. The female flower maturation index was lower in years when April temperatures were warm and when the prior year's acorn crop had been large. The overall size of the acorn crop was positively related to all three flower indices, with the maturation index playing the strongest role and the catkin and female flower indices being of similar strength based on their standardized effect sizes.

At the individual tree level (Figure 3b), both the catkin and female flower indices were greater in trees growing in warmer winter microclimates and with greater access to ground water as indicated by their XWP. The flower maturation index was, as predicted, inversely related to the catkin index. Ultimately, the acorn crop was positively related to all three flower indices, with the maturation index having the strongest effect and the catkin index the least based on their standardized effect sizes.

The finding that XWP and microclimate affect both catkins and female flowers at the individual tree level suggests that the two may be positively related, an unexpected result given self‐incompatibility, the adverse effect of catkins on flower maturation, and the potential for a life‐history tradeoff between a tree's investment in male and female flowers. In fact, the catkin and female flower indices were positively correlated (Pearson *r* = 0.44; *p* < 0.001), but resource availability among individuals can confound potential tradeoffs (van Noordwijk and de Jong 1986; Pease and Bull 1988). We addressed this problem by means of a linear regression of mean female flower abundance on mean catkin abundance, tree size (DBH), and three potential indices of resources: XWP, foliar N, and foliar P. Results confirmed a positive relationship between female flowers and catkins (effect size ± SE = 0.0667 ± 0.0246; *t*_67_ = 2.71, *p* = 0.009) controlling for all four other factors, none of which was significant (Table S3).

### 3.3 Acorn Production

At both the annual and individual tree levels, acorn production was significantly affected by all three flower indices (Figures 2b and 3b), explaining 95.4% of the variance in acorn production at the annual level (Table S1) and 48.2% at the individual tree level (Table S2). Based on their relative standardized effect sizes, the female flower maturation index was the most important in both cases, contributing 53.6% of the variance in annual acorn production and 42.4% of the variance in tree‐to‐tree acorn production. At the annual level, the catkin and female flower indices were nearly equal, contributing 24.8% (catkin index) and 21.6% (female flower index) to the variance in acorn production. At the individual level, the female flower index contributed 33.6% and the catkin index 24.0% to the variance in acorn production.

## 4 Discussion

### 4.1 Drivers of Flower Dynamics

At the individual tree level, microhabitat and access to ground water influenced both catkin and female flower abundance but did not directly influence flower maturation. As predicted, a smaller proportion of flowers matured into acorns from trees that produced more catkins. This suggests a tree's catkin production impedes its ability to successfully fertilize its own female flowers, a cost, plausibly, of self‐incompatibility. There was a positive relationship between a tree's catkin and female flower production, a result found even when controlling for resources. Individual trees do not trade off their overall investment in male and female flowers, as might be expected from life‐history theory. Instead, both male and female flower investment are related to a tree's access to resources, particularly ground water (Knops and Koenig 2012).

Results of the path analyses confirm the importance of resources to flower production. At the annual level, the significant effect of the prior year's acorn crop on flower maturation and the negative lag‐1 autocorrelation in the overall acorn crop discussed earlier are consistent with resource budget models, which propose that the resources required for successful seed production require more than a year to accumulate and that internal stored resource dynamics are key to variable reproduction (Isagi et al. 1997; Satake and Iwasa 2000; Pesendorfer et al. 2016).

The identity of critical resources and the degree to which *Q. lobata* trees are sensitive to a threshold of those resources prior to reproducing are not known, however. Our results indicate that access to groundwater affects flowering and productivity of individual *Q. lobata* , but the resources driving annual variability in acorn production by the population—masting behavior—remain unclear (Koenig et al. 2025).

Our results also support a key role for weather, both at the annual level and, although less easily measured, as a microclimatic factor affecting differences in flowering among trees. A relationship between weather and masting has been recognized for decades (Koenig 2021). There has, however, been considerably less work investigating the effects of weather on flowering and flower maturation (Cecich and Sullivan 1999), or on the links between weather and the physiology of reproduction in masting species (Bogdziewicz et al. 2025).

### 4.2 Mechanisms of Pollen Limitation

Pollen limitation is a key factor synchronizing reproduction among individuals in masting species (Isagi et al. 1997; Satake and Iwasa 2000; Crone et al. 2009) and has been shown to be important both empirically and experimentally in *Q. lobata* (Koenig et al. 2012; Pearse et al. 2015, 2026). There are at least three non‐mutually exclusive mechanisms by which pollen limitation can be expressed (Fleurot et al. 2024).

First are weather‐driven effects, including the pollination Moran effect and environmental vetoes discussed previously. Second is phenological synchrony, which differs from other weather effects because of its focus on timing rather than pollen abundance per se. Prior studies have found that flowering synchrony plays an important role in driving acorn production in *Q. lobata* , both at the individual tree level, where trees flowering during the peak of the season when the majority of other trees are flowering produce more acorns (Koenig et al. 2012), and at the annual level, where acorn production is greater in years when the population flowers more synchronously (Koenig et al. 2015; Pesendorfer et al. 2016).

A third means of pollen limitation is pollen coupling, an endogenous mechanism that focuses on female flowering being synchronized with external pollen from neighboring trees. Pollen coupling makes two predictions. First, it predicts a positive correlation between overall pollen availability and female flowering intensity, as both are key to overall acorn production (Rapp et al. 2013). Contrary to this prediction, we found no correlation between mean annual catkin abundance and mean annual female flowering based on our dataset (Pearson correlation *r* = −0.06, *N* = 10 years, *p* = 0.87). However, in the path analysis, both the catkin and female flower indices positively (and with nearly equal standardized effect size) affected the acorn crop, suggesting they have positive additive effects (Figure 2b).

This latter finding supports the prediction that investment in male and female flower production should be positively correlated at the annual level. Our test is limited, however, by the relatively few years for which we have female flower data and our assumption that overall pollen abundance can be inferred from our index of catkin production among our sample of trees. Despite notable attempts to measure pollen availability using remote Hirst‐type sensors (García‐Mozo et al. 2007; Ranta et al. 2008; Fleurot et al. 2024), external, non‐self pollen availability has yet to be quantified at the individual tree level. With new molecular techniques, this is likely to be possible in the near future.

A second prediction of pollen coupling is that there should be a negative lag‐1 autocorrelation in pollen abundance (Fleurot et al. 2024). At the annual level, the lag‐1 autocorrelation between mean catkin abundance was negative but not significant (−0.25; *p* = 0.46). At the individual tree level, however, 52 of 77 trees (67.5%) exhibited negative lag‐1 autocorrelations in their annual catkin index, significantly more than expected by chance (binomial test, *p* = 0.003).

Thus, evidence from flowering supports a role for pollen coupling as a driver of pollen limitation and reproductive synchronization in *Q. lobata* . Determining its relative importance compared to other mechanisms, including weather‐driven effects and phenological synchrony, is difficult as they act in combination to influence fruiting rates (Fleurot et al. 2024).

Our goal has been to identify the factors affecting flowering and pollen limitation. However, a large proportion of female flowers fail to mature even when provided with abundant pollen, indicating that other unknown factors affect female flower development (Cecich and Sullivan 1999; Pearse et al. 2015). Additional studies of flower fertilization and the successful development of fruits in oaks are desirable.

### 4.3 Flower Dynamics and Acorn Production

Historically, two main ways that flower dynamics drive acorn production have been recognized. At one end of the spectrum are “flower masting” species in which variable seed production is dependent on annual variation in flowering effort, a relationship that characterizes many perennial plants (Shibata et al. 2002; Bogdziewicz et al. 2025). At the other end are “fruit‐maturation masting” species in which variable pollination, fertilization, and fruit ripening are the main drivers of masting (Pearse et al. 2016).

Oaks have often been found to lie closer to the fruit maturation side of this dichotomy (Bogdziewicz, Szymkowiak, et al. 2019), but prior work on *Q. lobata* has indicated that both flowering effort and fruit maturation play a role in this species (Koenig et al. 2015; Pesendorfer et al. 2016). A recent study on a series of populations of *Q. petraea* in Europe found considerable variability in the role of flower dynamics with populations growing in relatively warm and seasonally moderate “soft” climates, such as the Mediterranean climate inhabited by *Q. lobata* , being driven by differences in female flower abundance rather than flower maturation (Fleurot et al. 2023).

Our results indicate, contrary to Fleurot et al.'s (2023) hypothesis regarding flower dynamics of trees growing in soft climates but in line with other studies of oaks (Bogdziewicz, Szymkowiak, et al. 2019), that female flower maturation plays a larger role than the number of female flowers produced in driving acorn production in *Q. lobata* , both at the annual and among‐tree levels. Both are important, however: *Q. lobata* is both a fruit‐maturation and flower masting species. Prior studies, however, have not considered a potential role for catkins, which are as important a driver of acorn production in *Q. lobata* at the annual level and contribute nearly a quarter to the variance in acorn production at the among‐tree level. Following current jargon, *Q. lobata* is not only partly a fruit‐maturation and female flower masting species, but also partly a male flower masting species.

## 5 Conclusion

Although both weather and resources have long been known to be important to masting behavior, our results provide new evidence regarding the complex ways they influence pollen limitation, flowering, and flower maturation, both among years and among trees. There are, however, many other potential factors that may affect flowering and fruiting behavior beyond those considered here (Knops et al. 2007; Sanchez‐Humanes et al. 2011). We have only begun to understand all the factors driving flower and acorn production in *Q. lobata* or other masting species.

Masting by oaks has previously been thought to be driven primarily by variability in female flower maturation, in contrast to beech ( *Fagus sylvatica* ), where masting is driven mainly by variability in female flower production (Bogdziewicz, Szymkowiak, et al. 2019; Journé et al. 2021). Our results support prior results indicating that both are important in *Q. lobata* . In addition, we found that male flower production (catkins) contributes significantly to differences in seed production. The factors affecting fruit crop size are clearly complex, non‐dichotomous, and mutually non‐exclusive (Kelly 2023). How and why the relative importance of flowering mechanisms varies, both intra‐ and inter‐specifically, remains a fruitful area for future research.

## Author Contributions

**Walter D. Koenig:** conceptualization (lead), data curation (equal), funding acquisition (lead), investigation (equal), methodology (equal), project administration (lead), writing – original draft (lead). **Ian S. Pearse:** conceptualization (equal), investigation (equal), methodology (equal), writing – review and editing (equal). **Mario B. Pesendorfer:** investigation (equal), methodology (equal), writing – review and editing (equal). **William J. Carmen:** investigation (equal), writing – review and editing (equal). **Johannes M. H. Knops:** conceptualization (equal), investigation (equal), writing – review and editing (equal).

## Funding

National Science Foundation, award numbers DEB‐0816691 and DEB‐1256394.

## Conflicts of Interest

The authors declare no conflicts of interest.

## Supporting information


**Table S1:** Statistical results of the path analysis for mean annual differences in *Quercus lobata* acorn production. Values listed are the unstandardized estimate ± standard error (SE); also listed are the *R* ^2^ values for each segment of the path. Significant relationships (*p* < 0.05) are highlighted and illustrated in Figure 1b.
**Table S2:** Statistical results of the path analysis for differences in mean acorn production among individual *Quercus lobata* . Values listed are the unstandardized estimate ± standard error (SE); also listed are the *R* ^2^ values for each path segment. Significant relationships (*p* < 0.05) are highlighted. DBH, Foliar N and Foliar P were not significant in any path and were excluded from the final model listed here and illustrated in Figure 2b.
**Table S3:** Results of a linear regression testing for the relationship between catkins and female flowers controlling for resources. Dependent variable is the mean female flower index. *N* = 70 trees.

## Data Availability

Data and code have been uploaded as Supporting Information. Data are also available at: doi: 10.5061/dryad.4qrfj6qst.
