# Supplementary material for: Flowering and Flower Maturation in Valley Oak Quercus lobata
Source: Ecol Evol. 2026 May 30;16(6):e73720. doi: 10.1002/ece3.73720 (PMC13240504; doi:10.1002/ece3.73720)
Supplement: Supplementary file 1 — Table S1: Statistical results of the path analysis for mean annual differences in Quercus lobata acorn production. Values listed are the unstandardized estimate ± standard error (SE); also listed are the R 2 values for each segment of the path. Significant relationships (p < 0.05) are highlighted and illustrated in Figure 1b. Table S2: Statistical results of the path analysis for differences in mean acorn production among individual Quercus lobata . Values listed are the unstandardized estimate ± standard error (SE); also listed are the R 2 values for each path segment. Significant relationships (p < 0.05) are highlighted. DBH, Foliar N and Foliar P were not significant in any path and were excluded from the final model listed here and illustrated in Figure 2b. Table S3: Results of a linear regression testing for the relationship between catkins and female flowers controlling for resources. Dependent variable is the mean female flower index. N = 70 trees. [file ECE3-16-e73720-s001.docx]

**Supplemental Material**

**Table S1** Statistical results of the path analysis for mean annual differences in *Q. lobata* acorn production. Values listed are the unstandardized estimate ± standard error (SE); also listed are the *R*^2^ values for each segment of the path. Significant relationships (*P* < 0.05) are highlighted and illustrated in Fig. 1b.

| Response variable | Explanatory variables | Estimate ± SE | *z*-value | *P*-value |
| --- | --- | --- | --- | --- |
| Mean catkin index  (*R*^2^ = 0.085) | |  |  |  |
|  | Winter rainfall | -0.001 ± 0.005 | -0.11 | 0.91 |
|  | Prior year’s acorn crop | 0.112 ± 0.121 | 0.92 | 0.36 |
| Mean female flower index  (*R*^2^ = 0.855) | |  |  |  |
|  | **Winter rainfall** | **-0.002 ± 0.000** | **-5.49** | **<0.001** |
|  | **Mean April temperature** | **0.012 ± 0.004** | **2.68** | **0.007** |
|  | Prior year’s acorn crop | 0.002 ± 0.007 | 0.33 | 0.74 |
| Mean flower maturation index  (*R*^2^ = 0.642) | |  |  |  |
|  | **Mean April temperature** | **-0.042 ± 0.015** | **-2.76** | **0.006** |
|  | Mean catkin index | -0.086 ± 0.056 | -1.52 | 0.13 |
|  | Mean female flower index | 0.433 ± 0.465 | 0.93 | 0.35 |
|  | **Prior year’s acorn crop** | **-0.062 ± 0.023** | **-2.71** | **0.007** |
| Acorn crop  (*R*^2^ = 0.954) | |  |  |  |
|  | **Mean catkin index** | **1.190 ± 0.183** | **6.50** | **<0.001** |
|  | **Mean female flower index** | **6.767 ± 1.101** | **6.15** | **<0.001** |
|  | **Mean flower maturation index** | **8.963 ± 0.645** | **13.89** | **<0.001** |

**Table S2** Statistical results of the path analysis for differences in mean acorn production among individual *Q. lobata*. Values listed are the unstandardized estimate ± standard error (SE); also listed are the *R*^2^ values for each path segment. Significant relationships (*P* < 0.05) are highlighted. DBH, Foliar N and Foliar P were not significant in any path and were excluded from the final model listed here and illustrated in Fig. 2b.

| Response variable | Explanatory variables | Estimate ± SE | *z*-value | *P*-value |
| --- | --- | --- | --- | --- |
| Mean catkin index  (*R*^2^ = 0.340) | |  |  |  |
|  | **Mean winter temp** | **0.117 ± 0.049** | **2.39** | **0.017** |
|  | **XWP** | **0.822 ± 0.136** | **6.05** | **<0.001** |
| Mean female flower index  (*R*^2^ = 0.177) | |  |  |  |
|  | **Mean winter temperature** | **0.034 ± 0.015** | **2.21** | **0.027** |
|  | Mean April temperature | -0.032 ± 0.021 | -1.51 | 0.13 |
|  | **XWP** | **0.103 ± 0.028** | **3.75** | **<0.001** |
| Mean flower maturation index  (*R*^2^ = 0.148) | |  |  |  |
|  | Mean April temperature | 0.012 ± 0.019 | 0.62 | 0.54 |
|  | XWP | 0.077 ± 0.044 | 1.75 | 0.08 |
|  | **Mean catkin index** | **-0.097 ± 0.032** | **-3.08** | **0.002** |
|  | Mean female flower index | 0.258 ± 0.154 | 1.67 | 0.10 |
| Acorn crop  (*R*^2^ = 0.482) |  |  |  |  |
|  | **Mean female flower index** | **5.097 ± 1.239** | **4.11** | **<0.001** |
|  | **Mean flower maturation index** | **4.864 ± 0.939** | **5.18** | **<0.001** |
|  | **Mean catkin index** | **0.653 ± 0.225** | **2.90** | **0.004** |

**Table S3** Results of a linear regression testing for the relationship between catkins and female flowers controlling for resources. Dependent variable is the mean female flower index. *N* = 70 trees.

| Independent variable | Estimate ±  standard error | *t-*value | *P-*value |
| --- | --- | --- | --- |
| Intercept | -0.1338 ± 0.1216 | -1.10 | 0.28 |
| Mean catkin index | 0.0667 ± 0.0246 | 2.71 | 0.009 |
| DBH | 0.0002 ± 0.0003 | 0.61 | 0.55 |
| XWP | 0.0322 ± 0.0345 | 0.93 | 0.35 |
| Foliar N | 0.0641 ± 0.0450 | 1.43 | 0.16 |
| Foliar P | -0.1960 ± 0.1652 | -1.19 | 0.24 |
